# Cross-Sectional Investigation of the Awareness and Practices of Food Safety among Food Service Catering Staff in Riyadh City Hospitals during the Coronavirus Pandemic

**DOI:** 10.3390/healthcare11081134

**Published:** 2023-04-14

**Authors:** Shaima Baker Alsultan, Shiekhah S. Allowaymi, Ghedeir M. Alshammari, Ali Alrasheed, Amro B. Hassan, Abdulmohsen Abdulaziz Alzobaa, Ahlam Bader Alqahtani, Mohammed Abdo Yahya

**Affiliations:** 1 Department of Food Science and Nutrition, College of Food and Agricultural Sciences, King Saud University, Riyadh 11451, Saudi Arabia; 2 Deputyship for Therapeutic Services, Ministry of Health, Riyadh 11345, Saudi Arabia; 3 First Health Cluster, Ministry of Health Riyadh, Riyadh 12769, Saudi Arabia; 4 King Fahad Medical City, Riyadh 11525, Saudi Arabia

**Keywords:** food safety awareness, food-handling practices, COVID-19

## Abstract

This study examines food safety awareness and practices among handlers in the food service sector at Riyadh City hospitals during the COVID-19 pandemic. Three hundred and fifteen (315) food service workers completed the entire questionnaire from five hospitals in Riyadh City between December 2020 and February 2021. The contributor’s respondents’ three-part questionnaire was divided according to general characteristics, food safety awareness, and food safety practices. The findings show that food handlers demonstrated good knowledge, techniques, and attitudes regarding maintaining food quality and ensuring food safety. Moreover, a significant positive correlation between food safety awareness and food safety practices was observed. Nevertheless, the correlation between the food handler’s knowledge and safe food handling was negative. In general, our findings revealed the significance of education and the regular training of food service staff to improve learning and ensure better and safer food-handling practices, which could contribute to applying food safety practices in hospitals.

## 1. Introduction

Food handling comprises a set of procedures to which food is exposed during the food production sequence until its final consumption, including receipt, storage, preparation, cooking, transportation, service, and presentation. To ensure food safety, food establishments should follow food safety control systems based on the principle of the HACCP system to control food safety hazards and prevent food contamination [1,2]. According to the World Health Organization (WHO), in January 2020, a new coronavirus (CoV) was discovered in China [3]. Coronaviruses (CoVs), which represent a large virus species, cause illnesses in the respiratory system that range from the common cold to more severe diseases, such as Middle East respiratory syndrome (MERS-CoV) and severe acute respiratory syndrome (SARS-CoV). The new coronavirus, which causes the illness 2019-nCOV or COVID-19, is a respiratory virus that spreads primarily from person to person through coughing and sneezing. Common signs of infection include a fever, a cough, and shortness of breath. In more severe cases, the contamination can cause acute respiratory syndrome, kidney failure, and even death [4]. According to the Saudi Arabian Ministry of Health statistics, on 26 July 2020, the number of coronavirus patients in Saudi Arabia reached 266,941. The number worldwide reached 16,055,909 and continues to increase.

The sanitary control of food in the food service sector is critical to reducing or mitigating outbreaks of endemic foodborne diseases. The Food and Drug Administration (FDA), the Centers for Disease Control and Prevention (CDC), and various food safety agencies agree that there is no proof to support the transmission of COVID-19 linked with food [5,6,7,8]. People consume food in public places, such as restaurants, hotels, and hospitals, and their food consumption is essential to daily life. To ensure the food safety provided to the community, food workers must be guided and trained in the necessary control measures for food safety and implement hygienic practices during food production [9].

Although no instances of food-service-related person-to-person transmission of COVID-19 have been reported, people working in the food service can be expected to be exposed to the risk of disease. Hence, this risk should not be ignored. It has been a challenge to ensure food safety and limit the spread of COVID-19 in the food service and retail sectors that serve perishable fresh food items that have passed through a series of steps, e.g., taking orders, preparing food, packing food, and delivering food to customers. In addition, hospital food services must follow all preventive measures to minimize the risk of food poisoning and ensure the safety of the meals they provide to patients [10]. The food service sector must strengthen its food safety management systems by following good health practices, including meeting the requirements for cleaning and public health and taking preventive measures in their dealings with suppliers, storage, distribution, transportation, and personal hygiene, in addition to all the standard conditions and activities necessary to maintain the cleanliness of the processing sites’ food. At each step, there is a possibility that food handlers who do not follow the appropriate precautionary measures might become a source of coronavirus spread by touching food or food surfaces [10].

So far, no recent published data exist to explore the work environment of the food service sector at Riyadh City hospitals. However, a solitary report has emerged, investigating knowledge, practice, and attitudes regarding food safety among food handlers preparing food for COVID-19 patients. Therefore, the current study was undertaken to assess the knowledge and food safety practices among food handlers in the food service sector at Riyadh City hospitals during the COVID-19 pandemic.

## 2. Materials and Methods

### 2.1. Study Design

This observational cross-sectional study was used to measure the research participants simultaneously and estimate the percentage of participants’ knowledge and attitudes. The questionnaire was performed following the Pen-and-Paper Personal Interviews (PAPI) method.

### 2.2. Questionnaire Design

A closed-ended questionnaire was designed based on pertinent and relevant questions from previously validated food safety questionnaires applied in similar studies [11,12,13,14,15,16,17,18] and following the guidance related to food safety in light of the coronavirus pandemic [19,20]. The questionnaire form contained twenty-eight questions, which were divided into three parts. First, seven questions on the demographic characteristics of each participant, such as their age, educational level, gender, nationality, and work experience, covered two questions on food safety training. In the second part, the questions focused on food handler knowledge and food safety practices. Finally, the last part of the questionnaire consisted of questions covering the knowledge of food handlers on COVID-19.

The questionnaire was validated through a pilot study amongst food safety professionals to verify the questionnaire’s accuracy and to strengthen the survey based on the feedback received. The questionnaire was later distributed manually to the food service employees in the 5 hospitals in Al-Riyadh. Participation was voluntary, and the respondents had the right to withdraw from the study. All food service staff in the selected five hospitals in Riyadh City were the target participants for this study. About 300 respondents agreed to participate in the research and completed the entire questionnaire. The questionnaires were distributed to five governmental hospitals, and staff responses were collected from December 2020 to February 2021. About 315 food service workers completed the entire questionnaire.

### 2.3. Statistical Analysis

The variables were summarized as descriptive statistics of frequency, percentage, and standard deviation. A multivariate analysis of variance (MANOVA) was used to examine the effects of the demographic variables. Statistical Package for the Social Sciences (SPSS), version 24 (IBM version 23.0, Armonk, NY, USA), was used to perform the quantitative analysis of the participants’ responses. The data are reported in numbers and percentages. In all cases, the cutoff for a significant effect was set at *p* < 0.05.

## 3. Results

### 3.1. Demographic Characteristics of the Food Handlers

Table 1 presents the demographic characteristics of study participants, with results showing that the majority of the employees were aged between 30 and 40 years (45.4%), followed by 18–29 (42.9%) and 41–50 years (9.5%), and the lowest number (2.2%) of employees were >51 years old. Regarding gender characteristics, 265 (84.1%) were males and 50 (15.9%) were females, with a male-to-female ratio of 5.3:1. Among the employees surveyed, most were non-Saudis. The majority (42.5%) were Indians, followed by 27.6% and 9.5% Bangladeshis and Filipinos. The majority of the study participants (48.6%) had more than four years of experience in catering services, and only 12.4% had less than a year of work experience. In total, 119 (37.8%), 45 (14.3%), 6 (1.9%), and 26 (8.3%) employees had secondary, bachelor’s, master’s, and diploma certificates, respectively. Food handlers surveyed in the present study belonged to varied job profiles, including chief cooks (10.8%), cleaners (21%), salad preparers (5.3%), meat cutters (1.6%), desert preparers (3.8%), and supervisors (4.1%), while more than half of them (53.3%) performed various roles in catering.

### 3.2. Current Awareness of COVID-19

Food handlers were assessed for their knowledge of the coronavirus’s symptoms and spread, as represented in Table 2. The majority of the participants (86%) were aware of all the main symptoms of the coronavirus. Similarly, 70% of the respondents knew that the virus’s incubation period was between 1 and 14 days, while around 20% did not know. Among all the food handlers, 53.7% knew that asymptomatic patients could transmit the virus, while 46.3% of respondents did not agree with this transmission mode.

### 3.3. Food Safety Knowledge of COVID-19 among Food Handlers

The catering staff was evaluated for their knowledge about personal hygiene and standard practices to control the coronavirus spread, and the results are summarized in Table 3. The majority of respondents (69.2%) agreed that effective cleaning, disinfection, and pest control could prevent the spread of coronavirus. Similarly, 83.8% of staff strongly agreed to wash their hands after entering the bathroom and touching their hair, face, nose, mouth, and ears. In addition, 97.2% of participants agreed that social distancing, washing hands frequently, and not touching their eyes and nose while working could thwart the coronavirus.

### 3.4. Knowledge of Handling and Storing Food among Food Handlers

Table 4 summarizes participants’ knowledge regarding handling food items during the pandemic to avoid cross-contamination. Results show that more than 90% of staff affirmed that hands should be washed and sanitized before handling food or eating and that using color-coded cutting boards can reduce cross-contamination. Although more than 60% of respondents knew that food handlers are a source of pathogenic bacteria and that thorough cooking can kill the coronavirus, a high percentage (39%) of participants gave negative responses. Overall, the catering staff demonstrated sufficient knowledge concerning handling and food storage. However, there is room for better handling of food items and preventing cross-contamination [21]. Other studies have reported similar results among hospital food handlers [22,23].

Regarding the overall knowledge level of respondents about food storage, the majority of respondents (86.3%) were aware that milk, meat, poultry, and eggs are perishable foods (Table 4). In total, 93.7% of respondents confirmed that cold foods should be kept at 41°F/5 °C or below, while 95.9% stated that hot foods should be kept at 140 °F/60 °C or above. Only half of the respondents knew the answer to the question about the normal temperature in a refrigerator (54.6%). The average temperature during freezing (49.8%), the minimum internal temperature of chicken when cooked (49.8%), the belief that food should be cooked until the center temperature reaches 74 °C to assure the death of pathogens (45.4%), the shelf life of refrigerated chicken (56.8%), and the shelf life of frozen chicken (17.8%). The results indicate a lack of knowledge on temperatures among food service staff, which agrees with various workers’ studies [14,24].

### 3.5. Knowledge about Coronavirus (COVID-19) among Food Handlers

Table 5 shows the knowledge of the coronavirus among the surveyed population of food handlers. Most of the respondents (67%) had no idea about the duration of the survival of coronavirus on glass surfaces. A relatively small percentage of participants (7.3%) reported that the virus could survive for 2 days, while 16.8% and 8.9% reported that the virus could survive for 5 and 9 days, respectively. The majority of respondents (66.7%) knew that the virus could survive on metallic, glass, and plastic surfaces. However, almost half of the respondents (48.7%) did not know about the chemical disinfectants that could prevent coronavirus, while 31.8% reported that ethanol, hydrogen peroxide, and/or sodium hypochlorite could be used for disinfection. Around 58% of the surveyed staff reported that the survival of the virus was dependent on the surface type, temperature, and environmental humidity. On the contrary, 24.1% of the staff had no idea.

Regarding the optimum time for washing hands with soap and water to limit the spread of coronavirus, only 43.5% of the respondents reported that their hands should be washed for 40 s. In summary, the respondents had little knowledge of the coronavirus and needed to be updated. In an Egyptian study by Elsherbiny and his colleagues conducted at hospitals to evaluate workers’ knowledge and practices, the majority of study participants (82.6%) did not know how long they should rub their hands during handwashing, and only 40.9% always washed their hands before handling food [25]. Authors reported the continued presence of coronaviruses (SARS, MERS, and COVID-19) on non-living surfaces such as metal, glass, and plastic for up to 9 days and maintained that these viruses could be effectively reduced by disinfecting surfaces with any of the following chemical disinfectants: ethanol alcohol (62–71%), sodium hypochlorite (1%), or hydrogen peroxide (5%) [9].

### 3.6. Knowledge and Practice of Food Safety

According to the data reported in Table 6, participants showed significant differences in their knowledge relating to coronaviruses according to nationality, education, work experience, and job role. Saudi participants reported significantly higher (*p* < 0.001) mean coronavirus knowledge as compared to non-Saudi participants (67.9 ± 12.1 vs. 52.6 ± 13.2). Participants who had master’s degrees reported significantly higher (*p* < 0.001) mean scores (69.2 ± 6.9) compared to participants with elementary, secondary, diploma, and other educational certificates. Furthermore, participants with more than 4 years of job experience also reported significantly higher (*p* < 0.001) mean scores (56.5 ± 14.5) compared to participants with less experience (48.9 ± 13.3). In addition, participants working as supervisors also reported the highest mean scores (61.5 ± 12.7) compared to cleaners and chief cooks (*p* < 0.05). Data showed that participants working as cleaners reported the lowest knowledge of coronavirus (49.9 ± 16.9) as compared to salad preparers and those working in other roles (*p* < 0.05). No significant differences in coronavirus knowledge were found according to age, gender, and food safety training courses.

Moreover, Table 6 shows significant differences in food safety practices according to age, qualification, and the extent of food safety training courses. Participants aged 30–40 years reported significantly lower mean scores on food safety practices than other age groups. Older participants, i.e., those over 50 years old, reported the highest mean score on food safety practices, i.e., 96.4 ± 9.4 (*p* < 0.05). This result agrees with a study conducted by [26] on food handlers in Calabria, Italy, which argued that food service staff differ in food safety practices according to their different age groups. However, contrary to this study [27], there was no relationship between hygiene practices and food handler ages. Participants with the lowest educational levels, e.g., at the primary level, reported the lowest mean score on food safety practices compared to participants with master’s, bachelor’s, and other academic certificates (*p* < 0.05). Similarly, the results show that participants who attended more than six food safety training courses significantly (*p* < 0.05) recorded higher scores on food safety practices (93.1 ± 14.2) as compared to participants who had not attended food safety training courses.

Similarly, the data of this study are consistent with the research conducted by [28,29,30], which found a strong association in the relationship between the education of food handlers and food safety practices. Others observed that trained food service staff achieved better scores on methods than untrained staff [31]. McIntyre et al. [32] reported that it is not necessarily true that a high level of education develops food safety practices among staff. No differences in food safety practices were found according to gender, nationality, job role, or job experience.

In this study, participants with an elementary education reported the lowest knowledge of food safety (74.6 ± 9.8) as compared to secondary and diploma certificate holders (*p* < 0.05). Similarly, studies found that there was a significant association between food safety knowledge and food service staff education levels. Moreover, the higher the food handlers’ education level, the better their food safety knowledge was [33,34,35,36]. On the contrary, however, a study found no significant differences in knowledge and practices and the education of food service staff [37]. Participants working as supervisors (84.6 ± 8.3) and in other roles (80.0 ± 11.0) reported significantly higher mean scores as compared to chief cooks, cleaners, and salad preparers (*p* < 0.05). This result contradicts another study conducted by [38], which showed that the job role did not improve the overall food safety knowledge assessment outcomes of hospital food handlers. No differences in food safety knowledge were found according to age, gender, nationality, job experience, or food safety training courses.

## 4. Discussion

The current study was considered to examine food safety awareness and practices among handlers in the food service sector at Riyadh City hospitals during the COVID-19 pandemic. Several questionnaires were designed to identify the food handlers’ origin and knowledge and training in food handling and storage. Moreover, the other part of the study covers the awareness of handling COVID-19 and practices of food handling with safety roles.

Concerning the safety training for the food handlers, our preliminary screening found that the majority (56.5%) of the team received one to three training courses, while 21.3% of food handlers did not undergo any training courses. Our findings indicate an emphasis on food safety training in hospitals in Al Riyadh. The American CDC identified five major risk factors in food services that are familiar sources of foodborne outbreaks: contaminated equipment, food from unsafe sources, improper holding times and temperatures, inadequate cooking, and poor personal hygiene. All these risk factors come from human error and behavior and can be prevented through proper safety training [39]. Studies found that food safety training improved knowledge of food safety [40,41,42]. One study on Korean restaurants confirmed the effectiveness of a training program, the results of which showed an improvement in food handlers’ knowledge and hygiene practices and reinforced the importance of handwashing before work [43]. Food handler training reduces food contamination and foodborne diseases [41].

Based on the handlers’ awareness of the coronavirus and its symptoms, we concluded that the majority of them in the target hospitals were aware of the symptoms. As stated by the WHO, the viruses are transmitted through droplets formed during coughing or sneezing. These droplets may enter the nose and mouth of other people [20]. A British study reported that infection symptoms of the emerging coronavirus (cough, fever, and loss of taste and smell) did not appear in 86 percent of those who tested positive, implying that those infected could spread the virus without symptoms appearing, emphasizing the importance of precautionary measures (wearing a mask, social distancing, cleaning, and disinfection procedures) to reduce the “silent” virus transmission [13,44]. Furthermore, another study stated that the risk of virus transmission via person-to-person contact, such as close contact with a patient or carrier, increased [45].

In this study, most of the participants (72.1%) knew that the virus does not spread through food, and 91.7% of food handlers agreed that the transmission of the coronavirus occurred through touching surfaces, shaking hands, and sneezing; these results agreed with a study conducted on food handlers working in food factories in Jordan based on this mode of transmission [13]. In addition, around 97.0% of food workers believed that inhaling big droplets while coughing and sneezing would be a significant factor in the spread of the SRS-CoV-2 virus, and 88.9% of food employees recognized that handshaking between coworkers would help transmit the virus. On the other hand, the workers had a limited understanding of other SRS-CoV-2 viral sources (such as contacting raw food; food packaging; contaminated surfaces; touching the nose, mouth, and eyes; and touching inanimate things (doorknobs, money, etc.)) [44]. Most of the catering staff were well-versed in the importance of sanitization in curbing the virus’s spread. In this study, the results revealed that 93.3% of employees stated that their temperature is taken before work begins on-site, 58% of respondents agreed that hand dryers are effective in killing the new coronavirus, and 50.8% indicated that spraying alcohol or chlorine all over the body is effective in killing the new coronavirus. These results contradict the guidance of the World Health Organization, which states the ineffectiveness of using hand dryers and spraying alcohol or chlorine all over the body to kill the new coronavirus [20]. Overall, most respondents had adequate knowledge about the symptoms and transmission of the coronavirus.

According to a recent study, one recommended preventive measure is social distancing. Social distancing is highly effective in all scenarios, such as airborne contamination when the microorganism remains viable in the air for an extended period, aerial transmission by coughing or sneezing, as well as direct or indirect physical contact (such as via contaminated surfaces, etc.) [46]. In total, 74.6–79% of catering staff fully agreed that maintaining personal hygiene and employing standard practices, such as washing hands after the disposal of waste and unfit food, keeping short and clean nails, wearing gloves and masks, and maintaining the appropriate distance between workers during food handling, could prove effective in controlling the spread of the virus. Strong agreement was recorded for 61% of respondents. In comparison, 27.3% agreed that, upon encountering suspicious or confirmed coronavirus cases among fellow workers, the central air conditioner must be cleaned and sterilized, and it can be turned on again after sanitation. Results demonstrate that the surveyed staff demonstrated in-depth knowledge of personal hygiene and standard practices to prevent the spread of coronavirus. In a study reported by university students in Jordan, the percentages of correct answers to “COVID-19 food-related attributes” were as follows: food cooking and storage (56.8%), personal hygiene (44.6%), and restaurant hygiene (36.9%) [44].

Regarding the food handlers’ practices, our preliminary findings stated that most participants (93.7%) reported covering their mouths and noses with a mask during food handling. The majority of the staff informed the correct way of washing hands, and 88.3% of respondents wash hands with warm water and soap and then wipe them dry. Moreover, about 94.6% of the surveyed staff reported wearing a cap or head covering when handling unwrapped foodstuff. In the case of the wound on the hands, 14.6% said to inform the manager, 7.6% of participants responded to covering the injury with a clean and impermeable bandage, and 7.9% of participants reported wearing gloves. The majority (69.8%) reported following all these protocols in the presence of wounds on their hands. In general, the staff stated acceptable food-handling practices. A similar result in a study conducted on restaurants in Vitoria, Spain, recorded good practices in personal hygiene (98.7%), uniform hygiene (93.3%), and keeping their hair completely covered during work (90.7%) [33]. A study conducted in Madinah hospitals indicated that nearly all employees (94.5%) always washed their hands before preparing food. Additionally, most employees (81%) wore gloves when handling food and always wore a mask (70.6%) and a head covering (82.2%) when preparing and serving food. In total, 70.6% of the employees reported that they knew the correct way to wash their hands [11]. Personal hygiene affects food hygiene as well as food quality and safety.

A correlation between the awareness of COVID-19, handling practices, and food safety knowledge of the handlers was also found in this study. The participants demonstrated that sufficient knowledge of the coronavirus tended to portray good food safety practices (R = 0.24, *p* < 0.001) and food safety knowledge (R = 0.27, *p* < 0.001). Moreover, those with higher food safety knowledge also showed good food safety practices (R = 0.39, *p* < 0.001). Similarly, other studies [11,33,47,48] describe a significant positive correlation between food safety knowledge and food safety practices. However, these results contrasted with those of another study that found that although food service employees had good knowledge of food safety, respondents seldom applied this knowledge when handling food [49,50].

In general, the findings of this study highlight the significance of education and the regular training of food service staff to improve learning and ensure better and safer food-handling practices, which could contribute to applying food safety and hygiene in hospitals, particularly in governmental hospitals.

## 5. Conclusions

The results of this survey show vital information regarding the food handlers’ level of knowledge, their attitudes and practices, the contamination of food samples, as well as an understanding of the coronavirus to ensure proper food safety. Food handlers demonstrated the use of sufficient knowledge, techniques, and attitudes with regard to maintaining food quality and ensuring food safety. However, there seems to be a pertinent need to educate food handlers about the coronavirus and its management to prevent the contamination or transfer of the virus. The findings of this study showed a significant positive correlation between food safety knowledge and food safety practices. However, there was no positive correlation between the food handler’s knowledge and safe food handling. Therefore, there is a need to evaluate training courses and to provide practical training for food handlers, following the mandates of the Ministry of Health.

## Figures and Tables

**Table 1:** The demographic characteristics of food service staff.

	Categories	Respondents	Percentage
Age Group	18–29	135	42.9
	30–40	143	45.4
	41–50	30	9.5
	>51	7	2.2
Gender	Male	265	84.1
	Female	50	15.9
Nationality	Saudi	30	9.5
	Egyptian	9	2.9
	Indian	134	42.5
	Bengali	87	27.6
	Filipino	30	9.5
	Sudanese	3	1.0
	Other nationalities	22	7.0
Job	Chief cook	34	10.8
	Cleaner	66	21.0
	Salad preparer	17	5.4
	Meat cutter	5	1.6
	Desert preparer	12	3.8
	Supervisor	13	4.1
	Any other role	168	53.3
Years of experience in food catering	<1 year	39	12.4
	2–3 years	123	39.0
	>4 years	153	48.6
Education	Elementary	92	29.2
	Diploma	26	8.3
	Secondary	119	37.8
	Bachelor’s	45	14.3
	Master's	6	1.9
	Other	27	8.6

Note: Data are presented as numbers and percentages.

**Table 2:** Food service staff information on the symptoms and transmission of COVID-19.

Questions	Categories	Respondents	Percentage
Symptoms of COVID-19 infection	High temperature	25	7.9
	Continuous cough	9	2.9
	Loss or change to sense of smell and taste	10	3.2
	All the above	271	86.0
COVID-19 incubation period (days)	1–3	7	2.2
	1–7	22	7.0
	1–14	222	70.5
	I do not know	64	20.3
COVID-19 is transmitted by a person without symptoms	Yes	169	53.7
	No	146	46.3
COVID-19 is transmitted through food	Yes	88	27.9
	No	277	72.1
COVID-19 is transmitted through touching surfaces, hand shaking, and sneezing	Yes	288	91.7
	No	26	8.3
Your temperature is taken before work starts on site	Yes	293	93.3
	No	21	6.7
Hand dryers are effective in killing the new COVID-19	Yes	182	58.0
	No	132	42.0
Spraying alcohol or chlorine all over your body kills the new COVID-19	Yes	160	50.8
	No	155	49.2

**Table 3:** Knowledge level of food service staff on hygiene and standard practices.

Questions	Categories	Respondents	Percentage
Effective cleaning, disinfection, and pest control prevent the spread of COVID-19	Strongly disagree	3	1.0
	Disagree	4	1.3
	Agree	90	28.6
	Strongly agree	218	69.2
You must wash your hands after entering the bathroom, touching hair, face, nose, mouth, and ears	Strongly disagree	3	1.0
	Disagree	2	0.6
	Agree	46	14.6
	Strongly agree	264	83.8
One of the correct practices to prevent COVID-19 is social distancing, washing hands frequently, and not touching eyes and nose while working	Strongly disagree	5	1.6
	Disagree	4	1.3
	Agree	43	13.7
	Strongly agree	263	83.5
You must wash your hands after disposal of waste and unfit food	Strongly disagree	5	1.6
	Disagree	1	0.3
	Agree	74	23.5
	Strongly agree	235	74.6
Fingernails should be kept short and clean	Strongly disagree	6	1.9
	Disagree	1	0.3
	Agree	72	22.9
	Strongly agree	236	74.9
You must wear gloves and a mask during food handling to prevent the spread of COVID-19.	Strongly disagree	7	2.2
	Disagree	2	0.6
	Agree	66	21.0
	Strongly agree	240	76.2
During food handling, there should be an appropriate distance between workers	Strongly disagree	2	0.6
	Disagree	3	1.0
	Agree	61	19.4
	Strongly agree	249	79.0
When suspicious or confirmed cases of COVID-19 are detected among workers, the central air conditioner must be cleaned and sterilized, and it can be turned on again after sanitation	Strongly disagree	14	4.4
	Disagree	23	7.3
	Agree	86	27.3
	Strongly agree	192	61.0

**Table 4:** (**a**) Food service staff information on the handling of food. (**b**) Food service staff information regarding food storage.

(**a**)			
**Questions**	**Categories**	**Respondents**	**Percentage**
You should wash and sanitize your hands before handling food or eating	Yes	311	98.7%
	No	4	1.3%
Food workers are a source of pathogenic microbes	Yes	190	60.3%
	No	125	39.7%
Using color-coded cutting boards can reduce cross-contamination	Yes	296	94.3%
	No	18	5.7%
Cooking thoroughly will kill the COVID-19	Yes	191	60.8%
	No	123	39.2%
(**b**)			
**Questions**	**Categories**	**Respondents**	**Percentage**
When chicken is cooked, the minimum internal temperature should be	55 °C	5	1.6%
	65 °C	33	10.5%
	75 °C	157	49.8%
	100 °C	45	14.3%
	I do not know	75	23.8%
Cold foods are kept at 5 °C or below	No	20	6.3%
	Yes	295	93.7%
Hot foods are kept at 60 °C or above	No	14	4.1%
	Yes	301	95.9%
The normal temperature in a refrigerator is	10–15 °C	11	3.5%
	5–10 °C	77	24.4%
	1–5 °C	172	54.6%
	Below 0 °C	3	1.0%
	I do not know	52	16.5%
To ensure killing pathogens, food should be cooked until the center reaches at least	64 °C	41	13.0%
	74 °C	143	45.4%
	100 °C	51	16.2%
	I do not know	80	25.4%
The normal temperature during freezing is	−20–−18 °C	157	49.8%
	−10 °C	70	22.2%
	0 °C	20	6.3%
	I do not know	68	21.6%
High-risk foods include	Milk	8	2.5%
	Meat	5	1.6%
	Poultry	17	5.4%
	Eggs	13	4.1%
	All of the above	272	86.3%
The shelf life (days) of refrigerated chicken is	1–3	179	56.8%
	4–7	55	17.5%
	>7	7	2.2%
	I do not know	74	23.5%
The shelf life (month) of frozen chicken is	5–6	166	52.7%
	7–8	22	7.0%
	9–12	56	17.8%
	I do not know	71	22.5%

**Table 5:** Food handlers’ knowledge about COVID-19.

Questions	Categories	Respondents	Percentage
COVID-19 remains on the glass surfaces up to	2 days	23	7.3%
	5 days	53	16.8%
	9 days	28	8.9%
	I do not know	211	67.0%
An example of surfaces on which COVID-19 remains	Metal	12	3.8%
	Glass	9	2.9%
	Plastic	13	4.1%
	All of the above	210	66.7%
	I do not know	71	22.5%
The chemical disinfectant that prevents COVID-19 is	Ethanol	57	18.2%
	Hydrogen peroxide	3	1.0%
	Sodium hypochlorite	1	3%
	All of the above	100	31.8%
	I do not know	153	48.7%
COVID-19 remains alive on surfaces depending on	Surface type	8	2.5%
	Temperature	25	7.9%
	Environmental humidity	22	7.0%
	All of the above	184	58.4%
	I do not know	76	24.1%
The best period by seconds to wash hands with soap and water to limit the spread of COVID-19 is	5 s	75	23.8%
	10 s	45	14.3%
	40 s	137	43.5%
	I do not know	58	18.4%
When you deal with unwrapped food, do you use a cap or wear a head covering?	Never	11	3.5%
	Sometimes	6	1.9%

**Table 6:** Respondents’ food safety knowledge, attitudes, and practices by their sociodemographic and employment characteristics.

	Coronavirus Knowledge		Practice		Food Safety Knowledge	
	Mean ± SD	*p* Value	Mean ± SD	*p* Value	Mean ± SD	*p* Value
Age (years)						
18–29	53.2 ± 13.5	0.193	88.0 ± 16.1	0.004	77.8 ± 13.7	0.236
30–40	53.8 ± 14.1		83.2 ± 20.3		77.1 ± 11.2	
41–50	58.9 ± 13.4		94.2 ± 14.2		81.9 ± 9.8	
>51	58.2 ± 13.9		96.4 ± 9.4		81.0 ± 10.3	
Gender						
Male	53.9 ± 13.5	0.593	87.1 ± 18.3	0.274	78.3 ± 12.7	0.199
Female	55.1 ± 15.4		84.0 ± 17.3		75.9 ± 9.4	
Nationality						
Saudi	67.9 ± 12.1	<0.001	85.8 ± 17.0	0.812	75.9 ± 9.6	0.326
Non-Saudi	52.6 ± 13.2		86.6 ± 18.3		78.1 ± 12.5	
What is your job?						
Chief cook	52.7 ± 13.4	0.033	91.9 ± 16.0	0.054	75.4 ± 9.8	0.005
Cleaner	49.9 ± 16.9		81.8 ± 20.8		74.1 ± 15.7	
Salad preparer	59.1 ± 14.5		88.2 ± 15.6		73.9 ± 10.9	
Meat cutter	50.8 ± 6.9		85.0 ± 22.4		77.1 ± 18.3	
Desert preparer	57.1 ± 14.1		81.3 ± 24.1		77.4 ± 8.9	
Supervisor	61.5 ± 12.7		96.2 ± 9.4		84.6 ± 8.3	
Any other role	54.9 ± 12.3		86.9 ± 17.3		80.0 ± 11.0	
How many years have you been working in food catering?						
<1 year	48.9 ± 13.3	0.003	89.7 ± 12.5	0.42	75.7 ± 9.8	0.312
2–3 years	52.8 ± 12.5		85.4 ± 17.5		77.5 ± 12.5	
>4 years	56.5 ± 14.5		86.8 ± 19.9		78.9 ± 12.6	
Qualification						
Elementary	53.3 ± 12.7	< 0.001	82.9 ± 17.4	0.044	74.6 ± 9.8	0.04
Diploma	56.3 ± 17.9		89.4 ± 14.4		78.4 ± 9.6	
Secondary	50.9 ± 13.0		85.5 ± 20.5		80.1 ± 14.9	
Bachelors	63.5 ± 13.7		91.1 ± 15.2		79.5 ± 10.6	
Master	69.2 ± 6.90		95.8 ± 10.2		80.2 ± 6.3	
Other	50.4 ± 9.1		91.7 ± 17.0		76.9 ± 10.6	
How many training food safety courses do you have?						
0	55.1 ± 13.8	0.066	82.8 ± 14.6	0.029	75.8 ± 11.4	0.454
1–3	52.7 ± 14.0		87.4 ± 17.9		78.5 ± 12.4	
4–5	54.1 ± 14.6		83.1 ± 26.6		79.0 ± 13.6	
>6	59.3 ± 10.6		93.1 ± 14.2		78.6 ± 11.7	

*p* value was determined by one-way ANOVA or independent sample, and values indicate statistically significant (*p* < 0.05).

## Data Availability

The datasets used and analyzed during the current study are available from the corresponding author upon reasonable request.
